# Supplementary material for: “Sun brings all things”: Sun and moon lore as biocultural knowledge on Aneityum island, Vanuatu
Source: PLoS One. 2025 Aug 1;20(8):e0327693. doi: 10.1371/journal.pone.0327693 (PMC12316303; doi:10.1371/journal.pone.0327693)
Supplement: S1 Appendix — Collection number refers to plant vouchers (herbarium specimens) used during interviews; initials and numbers refer to the collection numbers of the following collectors: AAM (Ashley A. McGuigan); GMP (Gregory M. Plunkett); MJB (Michael J. Balick). All plant specimens may be accessed in digital format at the CV Starr Virtual Herbarium, New York Botanical Garden, https://sweetgum.nybg.org/science/vh/. (DOCX) [file pone.0327693.s001.docx]

**Appendix 1**—Special plant uses related to Sun and Moon lore. Collection number refers to plant vouchers (herbarium specimens) used during interviews; initials and numbers refer to the collection numbers of the following collectors: AAM (Ashley A. McGuigan); GMP (Gregory M. Plunkett); MJB (Michael J. Balick). All plant specimens may be accessed in digital format at the CV Starr Virtual Herbarium, New York Botanical Garden, https://sweetgum.nybg.org/science/vh/.

*Names and/or usages that were elicited using vouchered photos of the collected plant, rather than an actual specimen.

| **Botanical species** | **Anejom̃ common name(s)** | **Usage** | **Collection number** |
| --- | --- | --- | --- |
| **(a) Plant materials that are sun-dried:** | | | |
| *Abutilon indicum* (L.) Sweet | *inhauam̃a* | In ancient times, this plant was used as a fiber to make skirts and rope. Take the stems, remove the leaves, ret the stems in sea water for a few weeks, sun dry the stems and then weave into rope or skirts. This plant is not much used for this purpose at the present time. | GMP 3560 |
| *Agave sisalana* Perrine | *korauri* | Put the leaf in seawater for two months to ret the fibers, then collect the fibrous strings and dry them in the sun and use them for weaving. In the past this plant fiber was used to make rope, but this is not common at present. | MJB 4974 |
| *Commersonia obliqua* Guymer | *nijma* | People make fiber out of the stem by taking the inner bark of this plant, drying it in the sun and using it to tie the beams of a house. | GMP 4775, GMP 3465* |
| *Dracaena* sp. nov | *nispeheñ* | This plant is used to make grass skirts for women. There are two different methods described. In the first, leaves are collected and torn in half [lengthwise], after which they are pleated. These are then dried in the sun by hanging them from the midrib. After they are dry, they are woven into a skirt. In the second method, tear the leaf down the center and then remove and discard the midrib. The soft part of the leaf blade is used to make the skirt by holding a piece of twine between one’s toes and under one’s armpit, and the length of this twine is woven with the leaf. These woven pieces are then dried in the sun for 2-3 days. The skirt can be thrown in the sea before drying to make it white. Bark of *inhau* (*Hibiscus tiliaceus*) is usually used as the twine. Retted strips of leaves, later sun dried, can also be used to make pillows. | GMP 3628, GMP 3628* |
| (a) *Ficus scabra* G. Forst, (b) *F. aspera* G. Forst. | *nauyerop̃* | To make a man's *kastom* belt, the stems of this plant are split and the outer bark peeled off, after which the end is torn to make a wide strap that can be tied. It is dried in indirect sunlight. | GMP 3487*, GMP 3487*, AAM 9* |
| *Freycinetia* cf. *reineckei* Warb. | *nameh* | The roots of this plant are used to make *nopoi*—a traditional trap used to catch fish and lobster. The outer bark of the roots is removed and sun-dried. The roots are then split into several pieces, and they are woven in an open fashion similar to a *noporapora*—a type of market basket fashioned from coconut leaflets. | GMP 4029 |
| *Freycinetia impavida* (Hombr. & Jacquinot ex Decne.) B.C. Stone | *nameh cedo* | The roots of this plant are used to make *nopoi*—a traditional trap used to catch fish and lobster. The outer bark of the roots is removed and sun-dried. The roots are then split into several pieces, and they are woven in an open fashion similar to a *noporapora*—a type of market basket fashioned from coconut leaflets. | GMP 4023 |
| *Hibiscus tiliaceus* L. | *intop̃hau* | This type of hibiscus can be used to make grass skirts. The outer bark of young shoots are peeled off, discarding the inner woody part. The bark strips are then scraped to remove the excess non-fibrous, green part, and the resulting strips are tied together and placed under stones in the sea for retting, where they sit for 5 days (but checked upon lon a daily basis). After the fibrous strips becomes soft, they are removed from underneath the stones, cleaned of any excess non-fibrous materials, and hung in the sun to dry for 3 days—this will bleach the fibers and give them a whitish color, at which point it can be woven into a skirt, neck garland, or hat, or used to decorate the handles of local baskets. | GMP 3202,  GMP 3202* |
| Melochia odorata L. f. | *nemula* | In making houses, the inner bark of this plant is dried in the sun and used to tie the house beams. | GMP 4757 |
| *Melodinus glaber* Turrill | *nuei* | This vine is very useful as a rope. (a) If the vine is dried up, it can be softened by soaking in seawater for 3-4 days. Afterwards, it is re-dried in the sun and will make a very strong rope used to lash parts of a traditional sleeping house. (b) The vine can also be collected fresh and heated over a fire to make it flexible and used for lashing while still warm. Another method of preparation is to place the vine in seawater for 3-5 days, at which point the outer bark will fall off and the inner portion of the vine will become flexible. At that point it can be used to tie parts of the house together. Exposure to the sun will dry it and bleach it a white color. The rope can also be prepared in the same way to lash together parts of a canoe. | (a) GMP 3589, (b) GMP 4762 |
| *Metroxylon warburgii* Becc. | *nuput* | The leaves of this palm are used for making thatch for roofs. Older leaves are collected, the pinnae are broken off, and the midribs are removed, after which the pinnae are woven onto a split stem of *Bambusa vulgaris* and dried in the sun. The midribs of this palm can be used to make local brooms (called *nisel an nuput*), or from the midribs of coconut leaves (called *nisel an neaig*). | GMP 3609 |
| *Morinda citrifolia* L. | (a) *nouruas*, (b) *nouras* | The inner, yellow-colored bark is used to dye clothes. This inner bark is scraped into a pot of water, then the cloth is immersed into it. After removing the cloth, it is dried in the sun, leaving the cloth with a yellow color. | (a) GMP 4774,  (b) GMP 3571* |
| *Myristica inutilis* A. Gray | *naijeg* | The prop roots of this species are naturally curved, allowing them to be used as a clothing hanger. Before the roots are used, the outer bark is peeled and dried all day in the sun. | GMP 4017 |
| *Nicotiana tabacum* L. | *tabake*, *tabac* | Yellowed, mature leaves from this plant are collected, rolled between one's hands, squeezed, and then dried in the sun for a day, after which they are hung in the kitchen near the place where fires are made; within a week, they will turn black, and are ready to be rolled inside a piece of paper and smoked. | MJB 4923 |
| (a, b) *Pipturus argenteus* (G. Forst.) Wedd., (c) *Nothocnide repanda* (Blume) Blume | *nelm̃ai*, *nelm̃ai apeñ* | The fibers of this plant are used to make fishing line, or for weaving small baskets and grass skirts and other things from this fiber. Tender stems are collected, and the outer bark is peeled off. This bark is then soaked (retted) in seawater for 1-2 weeks, with stones placed on top; this softens the bark and loosens the fibers, which can then be easily separated from the other tissues. Afterwards, the fibers are hung in the sun to dry and bleach. | (a) MJB 4863, MJB 4864 (b) AAM 17*, (c) MJB 5004 |
| *Pyrostegia venusta* (Ker Gawl.) Miers | *inwou itogau* | People use this vine as a local rope for lashing. The vine is collected and then heated over a fire to make it flexible. It is used to fasten things while still warm. Another method of preparation is to place the vine in seawater for 3-5 days, at which point the outer bark will fall off and the inner portion of the vine will become flexible. At that point, it can be used to lash parts of the house together, and the sun will dry it and bleach it a white color. This kind of rope can also be used to lash together parts of a canoe. | GMP 4758 |
| *Styphelia cymbulae* Spreng. | *naerek* | Branches of this plant are used to make local brooms. The branches are collected and dried in the sun, after which the leaves will fall off. The leafless branches are then tied together with a piece of *Pandanus* leaf (or any other vine that is handy). | GMP 3492 |
| *Trichospermum inmac* (Guillaumin) Burrett | *inmac* | The inner bark of this plant is used to make rope by peeling and removing the inner bark and then drying it in the sun. It can be used to tie the beams of a house. | GMP 3529* |
| *Urena lobata* L. | *namaka* | To make rope, the stems are cut, tied together in a bundle, and retted by placing them in the sea and weighing them down with rocks for about a week, after which they are removed from the sea, washed, and dried in the sun until the fibers bleach white. At this point, they can be used to make ropes or grass skirts. | MJB 4893 |
| *Zingiber zerumbet* (L.) Roscoe ex Sm. | *nijisei* | The leaf of this plant can be dried in the sun and then woven into skirts. | MJB 4912 |
| **(b) Plant-based medicines relating to the sun**: | | | |
|  |  |  |  |
| *Aceratium oppositifolium* DC. | *intoutau* | The leaves of this plant can be used to cast out bad spirits or heal headaches, fever, or any other kind of illness that modern medicine cannot fix. Four leaves from the top of this plant (*intoutau*) and three others (*netethae*, *nelmaha*, *inrowod*) are combined with 1/4 cup of water. The leaves are squeezed to make a juice and then poured into a piece of bamboo. The bamboo is then smashed open to release the mixture. This is done in the area where the sun sets, and the medicine must be released and drunk in the evening, before the sun sets. When a woman takes this medicine, she must drink half of the mixture and use the other half to wash her body, after which she must remain isolated from other people (except for those who helped wash her). | GMP 3635 |
| *Calophyllum neoebudicum* Guillaumin | *inpecei ielcei* | The leaf of this plant is used to treat a sick woman who was made ill from the male spirit known as *Paralelcei*. The leaves of this plant, mixed with other unspecified leaves, are tied together with a string and pounded until the juice is released. The juice is placed in a bamboo tube, and then the top of this tube is covered with wild cane leaves. This is then brought to the sick woman before sunset. Before she drinks the medicine, the bamboo tube is waved around her, then opened, and some of the medicine is poured on her head, after which she uses the liquid to wash her face and to drink. The bamboo is then broken and discarded before sunset. This practice tells the spirit to go away. Symptoms of this condition include miscarriage or prolonged menstrual period, or dreams that involve either the male spirit or snakes from the forest. | GMP 3584* |
| *Cordia dichotoma* G. Forst. | *incat iatou* | This plant can be used as a medicine to help prevent a person from getting a urinary tract infection (with painful urination) that results from being in the hot sun too long. During the heat of the day in the hot season, the inner bark from one branch of this tree is scraped it into 1 liter of water and drunk throughout the day | AAM 24* |
| *Ficus septica* Burm. f. | *nelmaha* | This plant is useful in treating headaches in women. Someone other than the patient must prepare this by breaking the top branch of *netethae* and removing its leaves for use. They are combined with the top leaves of the top branch of *nelmaha*, and both are chewed to produce a juice, which is then drunk. This is done when the sun is setting on the horizon. The woman gives the leftover fibers to the person who prepared the medicine, who then throws the fibers in the direction of the setting sun. | AAM 6,  AAM 21 |
| *Macaranga dioica* (G. Forst.) Müll. Arg. | *nihivai gaiyec* | To treat a headache resulting from being out in the sun too long, the outer bark of the stem of this tree is scraped off, and then scrapings from the inner bark are wrapped in a breadfruit leaf and placed in a fire for 15-20 minutes (but not in the hottest part of the fire, only in the flame). The juice is squeezed out of this bark while it is still warm and then rubbed all over the forehead and face to help alleviate the headache. | GMP 3213 |
| *Melastoma denticulatum* Labill. | *netedae* | This plant is useful in treating headaches in women. Someone other than the patient must prepare this by breaking the top branch of *netethae* and removing its leaves for use. They are combined with the top leaves of the top branch of *nelmaha*, and both are chewed to produce a juice, which is then drunk. This is done when the sun is setting on the horizon. The woman gives the leftover fibers to the person who prepared the medicine, who then throws the fibers in the direction of the setting sun. | AAM 27 |
| *Murraya paniculata* (L.) Jack | *neseu inm̃an* | To treat scabies, leaves of this plant are mashed together with grated coconut meat (endosperm), then this mixture is applied to the the body of the patient. After this, the person stands in the sun for 30 minutes, then immerses himself in fresh water to wash off this mixture. | GMP 4763 |
| *Scaevola taccada* (Gaertn.) Roxb. | *nanad copou* | This plant is used as a stimulant when a person goes to the garden early in the morning, before the sun comes up. Two young leaves are broken off and chewed., The liquid is swallowed, but the fiber is spat out. The effect of this is to allow the person to work harder and not feel tired while in the fields. It was noted that "a person can do the work of many people if he chews this." | GMP 3219 |
| *Volkameria inermis* L. | *neted woleg* | Used to treat scabies, one handful of leaves from this plant are harvested and taken to a place where rocks with holes are located close to the sea. The leaves are smashed and the sap squeezed into a pool of water in one of these holes. Then hot rocks are placed into the pool to warm it. People suffering from scabies bathe in this pool in the morning (only once a day) and then sit out in the sun. If they show signs of improvement, they repeat this treatment for 2-3 days, until the sores dry up. | GMP 3557 |
| **(c) Plants used as shade from the sun, for plants or humans**: | | | |
| *Ficus adenosperma* Miq. | *niditau* | The wood of this tree is used to make temporary houses (for example, to provide shade in a garden). | GMP 3636 |
| *Ficus obliqua* G. Forst. | *nerere* | This tree is good for shade. | GMP 3694 |
| *Heliotropium arboreum* (Blanco) Mabb. | *nanad op̃a* | This coastal tree is a good to lay under because it provides shade. | GMP 3542 |
| *Operculina turpethum* (L.) Silva Manso | *unmowad upikad, inmowad up̃ikod* | This vine forms a thick canopy, so some people plant it around their houses, near trees that do not give much shade, to reduce the intensity of the sun on the house and thus keep the temperature lower. | MJB 5009 |
| *Schleinitzia insularum* (Guill.) Burkart | *naerum̃an* | This plant is considered "nambawan" (the best) for shade. | GMP 3576 |
| *Tabernaemontana pandacaqui* Poir. | *inmadidi* | This is known as a good source of shade for people who are walking along the coast. | GMP 4768 |
| **(d) Plant-based foodways relating to the sun**: | | | |
| *Cordyline fruticosa* (L.) A. Chev. | *inrowod* | The roots of this plant can be cooked in an earth oven. These need to be cooked for 2 days or 2 nights, like a yam. Once cooked, they can be stored for 6 months. In ancient times they were eaten when there were no other sources of food. This food is said to be able to sustain a person for one day, and if it is eaten in the morning, the person will not be hungry until sunset. Today, people eat this plant at festivals, as it is no longer typically used as a famine food. | GMP 3526 |
| *Tacca leontopetaloides* (L.) Kuntze | *masoa* | This plant was said to have been brought in by the early missionaries, used to generate starch for their clothes and grown as a crop for export to England. To process, the roots are put in a bowl, water is added, and the roots are soaked for 1 day and 1 night. Then the water is poured off and the starch is retained. The root can also be used as a food crop as well. In one method, it is mashed and then dried in the sun, to be kept until needed. It can then be prepared as a kind of lap-lap but cooked in a frying pan on an open fire. Alternatively, the fresh tuber can be grated into a dish and washed with water until the starch settles; the water is poured off, the remaining starch is dried in the sun and then pounded and cooked with coconut milk. | (a) MJB 4915  (b) GMP 3438* |
| *Heliconia indica* Lam. | *inmehei* | The leaves of this plant are used to construct an airtight container to ferment *namarai* in an underground pit. | MJB 4986 (Fig. 5) |
| **(e) Plants used in sun-related weather magic and ritual**: | | | |
| *Curculigo orchioides* Gaertn. | *naligaj* | This low plant is used in Aneityum weather magic, to influence the wind and/or sun. It also has other magical uses (not relating to the sun) and the roots can be used as a famine food. | MJB 4985 |
| *Scaevola cylindrica* Schltr. & K. Krause | *nanad itohou* | In ancient times, people would collect the sapwood of this tree together with *nijiga* (*Vaccinium macgillivrayi* Seem.) from the West side of Aneityum and cross the mountain to the East side. When they got to the red-clay mountain known as Nedun Necdaduin Cap, they would burn these two plants as an offering to the spirits to get more sun anbd less rain so that their gardens would grow well. This is also part of the Namlainhas ritual. | GMP 3456 |
| *Vaccinium macgillivrayi* Seem. | *nijiga, nedun nijiga* | This shrub is burned in Aneityum weather magic rituals, used to influence wind and/or sun. In the sun ritual, it is used together with *nanad itohou.* | GMP 3591 |
| *Heliotropium arboreum* (Blanco) Mabb. | *nedun nijiga, nedun nanad opã* | Plant burned in Aneityum weather magic ritual, to affect wind and/or sun. |  |
| *Blepharoglossum condylobulbon* (Rchb. f.) L. Li | *naepomyiv* | Children often take taut leaves of this plant and blow across the surface to make a whistle sound. Older folks say this blowing invited the rain and was used as needed. | GMP 4113 |
| *Heliotropium foertherianum* Diane & Hilger | *nanad op̃a* | This plant is used in the *Namlainhas* ceremony to call a burning sun. | GMP 3542 |
| *Piper latifolium* L.f. | *incaceñ upni* | A kind of (“good”) domesticated kava, used in rituals and ceremonies, including weather magic, as well as for daily consumption. |  |
| *Piper methysticum* G. Forst. | *incaceñ, kava* | This is the true kava plant, used in the *tam̃ava* ceremony, when the chief of a certain crop ritually spits kava to “stamp” or “seal” the daily reports of gardeners on their crops. This sends a report on the gardens to the sun, from which the report is transferred to the moon, then to *Iplu-Halu Comñomoi* (the Pleiades), and finally back to the *graon* (“earth”), who can adjust the weather to keep the crops healthy. When a gardener knows specifically what weather changes are needed, *tam̃ava* can also be made intentionally by weather magic practitioners to the sun, who then makes a report to the tribe’s spirits. |  |
